# Impact of the COVID-19 Pandemic on Histopathological Cancer Diagnostics in Lower Silesia: A Comparative Analysis of Prostate, Breast, and Colorectal Cancer from 2018 to 2022

**DOI:** 10.3390/cancers17010134

**Published:** 2025-01-03

**Authors:** Danuta Szkudlarek, Katarzyna Kalinowska, Benita Wiatrak

**Affiliations:** 1 Pathology Department, Provincial Hospital Center of the Jelenia Góra Valley, Ogińskiego 6, 58-506 Jelenia Góra, Poland; 2 Department and Clinic of Pulmonology and Lung Cancers, Wroclaw Medical University, ul. Grabiszynska 105, 53-439 Wroclaw, Poland; 3 Department of Pharmacology, Faculty of Medicine, Wrocław Medical University, Mikulicza-Radeckiego 2, 50-345 Wrocław, Poland

**Keywords:** COVID-19, breast cancer, colorectal cancer, prostate cancer

## Abstract

Background/Objective: The COVID-19 pandemic significantly disrupted healthcare systems worldwide including cancer diagnostics. This study aimed to assess the impact of the pandemic on histopathological cancer diagnostics in Lower Silesia, Poland, specifically focusing on prostate, breast, and colorectal cancer cases from 2018 to 2022. The objective was to evaluate diagnostic volumes and trends before, during, and after the pandemic and to understand the effect of healthcare disruptions on cancer detection. Methods: Histopathological and cytological data were collected from multiple laboratories across Lower Silesia. Samples were categorized into three periods: pre-pandemic (January 2018–February 2020), pandemic (March 2020–May 2022), and post-pandemic (June–December 2022). Statistical analyses included comparisons of diagnostic volumes and positive diagnoses across these periods. Results: A significant reduction in the number of histopathological examinations occurred during the pandemic, particularly during its early phase. This decline was accompanied by a higher frequency of positive cancer diagnoses, suggesting the prioritization of high-risk cases. Post-pandemic, diagnostic activity showed partial recovery, though it remained below the pre-pandemic levels, with notable differences across cancer types. Conclusions: The COVID-19 pandemic significantly disrupted cancer diagnostics in Lower Silesia, delaying detection and highlighting healthcare system vulnerabilities. These findings underscore the importance of resilient healthcare systems that can ensure the continuity of essential diagnostic services and address inequalities in access to care during crises.

## Simple Summary

The COVID-19 pandemic caused significant disruptions to healthcare systems including cancer diagnostics. This study examines how the pandemic affected the diagnosis of prostate, breast, and colorectal cancers in Lower Silesia, Poland, between 2018 and 2022. By analyzing data from histopathology laboratories, we identified a sharp decline in the number of diagnostic tests during the pandemic and a slower recovery afterward. Interestingly, the frequency of positive diagnoses increased, suggesting a focus on higher-risk cases. These findings highlight the need for healthcare systems to be better prepared for crises to ensure timely and equitable access to cancer diagnostics. This research provides insights into the challenges faced during the pandemic and offers valuable data for planning improvements in healthcare systems to minimize disruptions in the future.

## 1. Introduction

Malignant tumors are among the most serious global health issues, representing a significant cause of death and impacting the patients’ quality of life [1,2]. In recent years, there has been a noticeable increase in cancer incidence, driven by genetic and environmental factors as well as population aging and improved access to diagnostics. Breast cancer, prostate cancer, and colorectal cancers are among the most frequently diagnosed malignant tumors [3], highlighting their importance in epidemiological and clinical research. Breast cancer, the most common malignancy in women, accounts for a substantial number of cancer cases and deaths [1]. Efforts in both developed and developing countries aimed at early detection and treatment have contributed to improved survival rates [2]. Similarly, prostate cancer, the most frequently diagnosed malignancy in men, plays a key role in the overall cancer burden [1]. Colorectal cancers, which include cancers of both the colon and rectum, rank third in terms of incidence in both sexes, with their diagnosis and treatment constituting a crucial element of healthcare [1,3].

The COVID-19 pandemic, which began at the end of 2019, has significantly impacted healthcare systems worldwide including cancer diagnostics and treatment [4]. Global analyses including studies conducted in the USA, Europe, and Poland clearly indicate that delays in cancer diagnostics and reduced screening during the COVID-19 pandemic have led to an increase in cancers diagnosed at more advanced stages [4,5,6]. The implementation of pandemic-related restrictions, such as lockdowns, limited access to medical facilities, and patient fears of infection, resulted in delays in cancer diagnosis and treatment. Studies have shown that the number of screening and diagnostic tests decreased significantly in many countries during the pandemic, potentially leading to later cancer detection and negatively affecting patient outcomes. An analysis of data from 2018 to 2022 allows for an evaluation of the pandemic’s impact on cancer diagnosis rates and comparisons between the periods before, during, and after the peak restrictions.

In a regional context, Lower Silesia, as one of the larger regions of Poland, plays a significant role in the national healthcare system. An analysis of cancer epidemiology data from this region can provide valuable insights into the pandemic’s effects on cancer diagnostics and the effectiveness of the implemented health strategies [4]. Histopathological examinations play a crucial role in cancer diagnostics, allowing for the precise assessment of tumor type, stage, and morphological characteristics. Detailed analysis of microscopic specimens not only confirms the presence of cancer, but also identifies other non-cancerous pathological changes that may coexist in the examined tissues [7]. In the context of the COVID-19 pandemic, the analysis of diagnosed cases before, during, and after the pandemic provides important data on the impact of the global health crisis on cancer diagnostic systems.

The aim of this study was to present the results of histopathological examinations conducted in Lower Silesia during 2018–2022 with a particular emphasis on cases of breast cancer, prostate cancer, and colorectal cancer or adenoma. The analysis also included an assessment of the number of other non-cancerous changes in the examined samples. This study evaluated the impact of the pandemic on cancer diagnostics in Lower Silesia, comparing three critical time periods: before the pandemic (January 2018–February 2020), during the pandemic (March 2020–May 2022), and after the pandemic (June–December 2022). These results will provide valuable information on cancer incidence dynamics and the effectiveness of the diagnostic system in the region in the context of the pandemic, contributing to a better understanding of the population’s healthcare needs and more effective planning of preventive and therapeutic measures in the future.

## 2. Materials and Methods

### 2.1. Data Collation

In this study, data were sourced from histopathology and cytology laboratories located in pathology departments that analyzed samples collected from various hospitals and clinics across Lower Silesia. The data came from provincial, district, university hospitals as well as private medical practices. The analysis focused on the total number of histopathological examinations conducted on samples obtained from organs such as the colon, breast, and prostate. All results were assessed for the presence or absence of cancer, distinguishing between positive and negative findings, and were evaluated monthly over the period from 2018 to 2022. The study’s results were collected from hospitals across Lower Silesia including both large urban hospitals and smaller district hospitals to ensure comprehensive data coverage from all facilities except the Lower Silesian Oncology Center (DCO), which was closed during the initial phase of the pandemic. There was no grouping of hospitals based on size; instead, the focus was on obtaining results from a diverse range of healthcare facilities within the region.

The data analysis was conducted based on the number of tests performed during the pandemic as well as before and after the pandemic. Additionally, the impact of the first wave of the pandemic (covering the four months from March to June 2020) on the number of diagnosed cases was compared. The statistical analysis included only results coded according to the International Classification of Diseases, version 10 (ICD-10).

### 2.2. Study Population and Variables

The study encompassed all histopathological test results from patients aged 18 years and older excluding the pediatric population from the Lower Silesia region. Data from the Lower Silesian Oncology Center were excluded due to the hospital’s closure during the first wave of the pandemic. The analyzed dataset covered the period from January 2018 to December 2022. Only patients who had no prior cancer diagnosis were included in the study. Cancer diagnoses were classified based on location: (1) breast cancer (ICD-10: C50) analyzed only in women; (2) prostate cancer (ICD-10: C61) analyzed in men; (3) colorectal cancer (ICD-10: C18-C20) analyzed in both sexes. This classification ensured the accurate coding of cancer types based on their anatomical location.

### 2.3. Statistical Analyses

Statistical analyses were conducted using Statistica 14.1 software (TIBCO Software Inc., Palo Alto, CA, USA). A *p*-value of <0.05 was considered statistically significant. A Kruskal–Wallis test with post hoc analysis was applied to compare the total monthly number of tests and the cancer or adenoma detection rate across the analyzed periods before, during, and after the pandemic.

## 3. Results

The obtained data indicated differences in the number of histopathological tests performed and cancer diagnoses before, during, and after the pandemic. After converting the number of tests and diagnoses performed into monthly average values, notable trends were observed, which are reflected in the comparative analysis of the histopathological test volumes across three periods: before the pandemic (January 2018–February 2020), during the pandemic (March 2020–May 2022), and after the pandemic (June–December 2022).

### 3.1. Prostate Cancer

The examination of data related to the number of biopsies and submitted materials for the histopathological assessment of prostate cancer cases from 2018 to 2022 as well as the overall number of diagnosed tumors including non-cancerous changes revealed significant shifts over different time periods, particularly in light of the COVID-19 pandemic.

**Figure 1:**
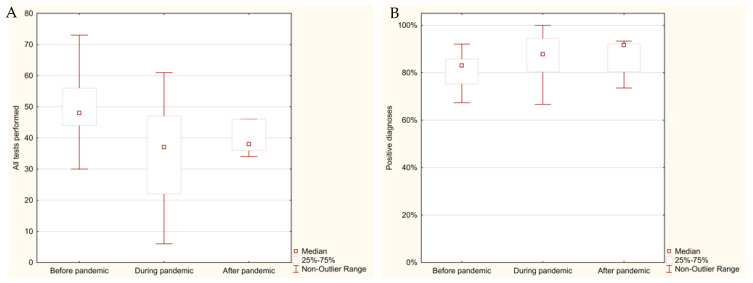
Distribution of monthly histopathological tests for prostate cancer (2018–2022) represented as a box plot: (**A**) total number of tests performed; (**B**) frequency of positive prostate cancer diagnosis.

Table 1 and Figure 1 provide a detailed overview of the number of tests conducted for prostate cancer, and the positive diagnoses before, during, and after the COVID-19 pandemic. The data showed significant fluctuations in the number of tests and diagnoses throughout these periods.

Before the pandemic, between January 2018 and February 2020, an average of 49.85 histopathological tests were performed per month, resulting in 40.46 positive prostate cancer diagnoses. The frequency of positive diagnoses during this time was 81.2%.

During the pandemic, from March 2020 to May 2022, the average number of monthly tests decreased significantly to 35.11, with 30.22 positive diagnoses. Statistical analysis confirmed that this reduction in the number of tests compared to the pre-pandemic period was highly significant (*p* = 0.002). Despite the reduction in overall testing, the frequency of positive diagnoses increased to 86.0%. The most pronounced decline occurred during the first wave of the pandemic (March–June 2020), when the number of tests dropped to just 17 per month, of which 14.75 were positive for prostate cancer, corresponding to a frequency of 86.8%.

**Figure 2:**
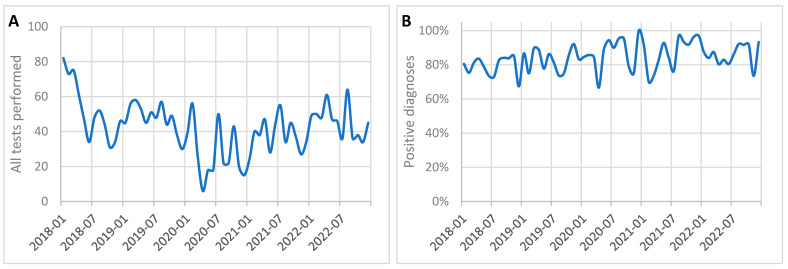
Monthly trends in histopathological tests for prostate cancer performed between 2018 and 2022: (**A**) total number of tests performed; (**B**) frequency of positive prostate cancer diagnosis.

The data presented in Figure 2 illustrate these trends in greater detail, showing month-by-month variations in the total number of tests and the proportion of positive diagnoses. The number of tests performed declined sharply at the onset of the pandemic in March 2020, reaching its lowest point during the first wave. The data also showed a gradual recovery in test volumes following the pandemic, with testing levels stabilizing by late 2022. However, statistical comparisons of the number of tests performed showed no significant differences between the pre-pandemic and post-pandemic periods (*p* = 0.5387) or between the pandemic and post-pandemic periods (*p* = 1.0).

Following the pandemic, between June and December 2022, the number of tests partially rebounded to an average of 42.71 per month, with 37.43 positive diagnoses. During this period, the frequency of positive diagnoses rose further to 87.6%.

The frequency of positive prostate cancer diagnoses increased slightly during the pandemic and post-pandemic periods, despite a significant reduction in the number of tests performed. Statistical comparisons of the number of positive diagnoses revealed significant differences between the pre-pandemic and pandemic periods (*p* = 0.0329) but not between the pre-pandemic and post-pandemic periods (*p* = 0.2351) or between the pandemic and post-pandemic periods (*p* = 1.0).

These findings highlight the impact of the COVID-19 pandemic on prostate cancer diagnostics, with a marked reduction in testing during the pandemic’s early phases, followed by a partial recovery post-pandemic. However, the relatively stable frequency of positive diagnoses suggests that the prioritization of suspected cancer cases during the pandemic may have mitigated its potential impact on diagnostic outcomes. The increase in the frequency of positive diagnoses suggests that the prioritization of suspected cancer cases during the pandemic may have mitigated its potential impact on diagnostic outcomes, despite the reduction in the number of tests performed.

### 3.2. Breast Cancer

**Figure 3:**
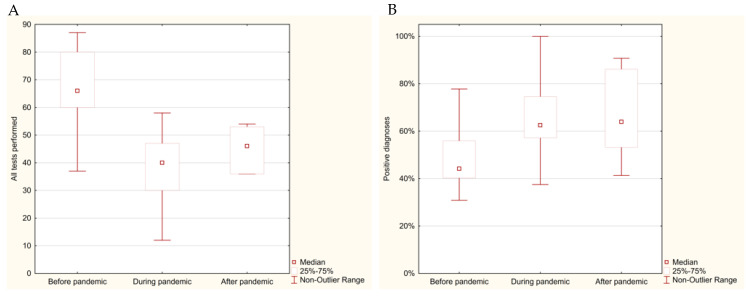
Distribution of monthly histopathological tests for breast cancer (2018–2022) represented as a box plot: (**A**) total number of tests performed; (**B**) frequency of positive breast cancer diagnosis.

The study analyzed the histopathological data on breast cancer from 2018 to 2022, focusing on the number of tests conducted, the frequency of diagnoses, and the impact of the COVID-19 pandemic. Detailed data on the number of tests and diagnoses across the pre-pandemic, pandemic, and post-pandemic periods are presented in Table 2 and Figure 3.

Before the pandemic, from January 2018 to February 2020, the number of histopathological tests for breast cancer was relatively stable, averaging 67.23 per month, with 31.50 positive diagnoses. The frequency of positive diagnoses during this period was 46.85%. Statistical analysis showed a significant difference in the number of tests conducted before the pandemic compared to during the pandemic with a *p*-value < 0.001, confirming a substantial reduction in diagnostic activity.

During the pandemic, from March 2020 to May 2022, the number of tests decreased dramatically to an average of 37.93 per month, while positive diagnoses dropped to 24.74 per month. The most severe decline occurred during the first wave of the pandemic (March–June 2020), with monthly averages falling to 24.50 tests and 15.75 diagnoses. Despite fewer tests being conducted, the frequency of positive diagnoses increased significantly to 65.23% (*p* < 0.001 when compared to pre-pandemic levels).

After the pandemic, from June to December 2022, there was a partial recovery in diagnostic activity, with the number of tests rising to 44.71 per month and positive diagnoses increasing to 29.29. The comparison of the pre-pandemic and post-pandemic periods showed that after the pandemic, the number of tests performed remained significantly lower (*p* = 0.008). The frequency of positive diagnoses remained relatively high at 65.50%, consistent with the trends observed during the pandemic.

**Figure 4:**
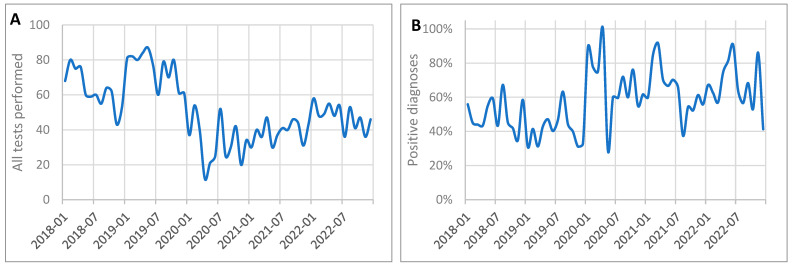
Monthly trends in histopathological tests for breast cancer performed between 2018 and 2022: (**A**) total number of tests performed; (**B**) frequency of positive breast cancer diagnosis.

Data from Figure 4 illustrate the trends in histopathological testing and diagnosis rates on a monthly basis. The number of tests dropped sharply in early 2020, reaching its lowest point during the first wave of the pandemic, and gradually recovered through 2022. Despite fluctuations in test volumes, the frequency of positive diagnoses remained higher during and after the pandemic compared to the pre-pandemic levels.

The results showed a significant decline in the number of tests during the pandemic, with a partial recovery afterward. Despite the reduced testing volume, the frequency of positive diagnoses increased during and after the pandemic compared to the pre-pandemic levels, highlighting notable shifts in diagnostic patterns over the studied period.

### 3.3. Colorectal Cancer and Adenoma

This study analyzed the histopathological data on colorectal cancer and adenoma from 2018 to 2022, focusing on the number of tests conducted, the frequency of diagnoses, and the impact of the COVID-19 pandemic.

Before the pandemic, from January 2018 to February 2020, the average number of histopathological examinations for colorectal conditions was 305.73 per month, with 66.35 cases of colorectal cancer and 146.50 adenomas diagnosed monthly. The frequency of cancer or adenoma diagnoses during this period was 69.62%.

**Figure 5:**
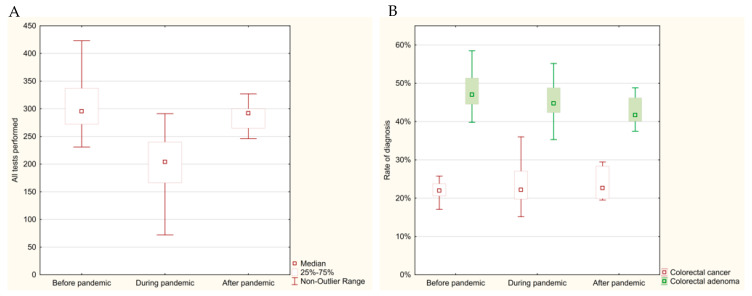
Distribution of monthly histopathological tests for colorectal cancer (2018–2022) represented as a box plot: (**A**) total number of tests performed; (**B**) frequency of diagnosis of colorectal cancer of adenoma.

During the pandemic, from March 2020 to May 2022, the number of examinations dropped significantly to 197.85 per month, with 44.04 colorectal cancer cases and 89.74 adenomas diagnosed monthly. The most severe decline occurred during the first wave of the pandemic (March–June 2020), when monthly averages fell to 107.50 examinations, 25.25 colorectal cancer cases, and 50.50 adenomas. Across the entire pandemic period, the frequency of cancer or adenoma diagnoses was 67.62%, slightly lower than before the pandemic. During the first wave, however, this frequency increased slightly to 70.47%. Detailed data are presented in Table 3 and Figure 5. The comparison of the number of diagnoses before, during, and after the pandemic did not reveal any significant statistical differences (*p* > 0.05).

In 2022, after the pandemic subsided, the diagnostic activity began to recover. The number of histopathological examinations increased to 287.86 per month, with 67.57 cases of colorectal cancer and 122.71 adenomas diagnosed monthly. Comparisons between the pandemic and post-pandemic periods showed a significant increase in the number of examinations (*p* = 0.0043).

**Figure 6:**
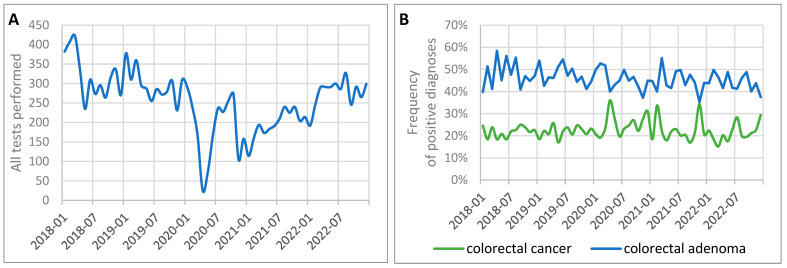
Monthly trends in histopathological tests for colorectal cancer performed between 2018 and 2022: (**A**) total number of tests performed; (**B**) frequency of diagnosis of colorectal cancer or adenoma.

Data from Figure 6 illustrate the significant fluctuations in monthly diagnostic activity for colorectal cancer and adenomas during the study period. The number of tests declined sharply at the onset of the pandemic in 2020, reaching its lowest point in April 2020, when only nine colorectal cancer cases were diagnosed. Diagnostic activity partially recovered in 2021 and further improved in 2022, reflecting a gradual return to normal healthcare operations and efforts to address the diagnostic backlog caused by the pandemic.

The COVID-19 pandemic significantly disrupted diagnostic activity for colorectal conditions, with a notable decline in the number of histopathological examinations and diagnoses of colorectal cancer and adenomas. Although diagnostic activity began to recover in 2022, it has not fully returned to its pre-pandemic levels. The frequency of cancer or adenoma diagnoses remained relatively stable across the study periods.

## 4. Discussion

The COVID-19 pandemic had a profound impact on cancer care worldwide [4,8]. In this paper, we discussed the changes in diagnostics, treatment, and overall health outcomes for cancer patients during the pandemic, referencing the latest scientific research. Specifically, we focused on delays in diagnosis and treatment, healthcare system adaptations, and the widening disparities in access to cancer care. Additionally, we highlighted the role of screening tests and the necessity of developing new, rapid, and minimally invasive diagnostic methods that can be used outside hospitals. The results suggest that the pandemic significantly worsened cancer treatment outcomes, requiring further research and strengthening healthcare systems in the face of future crises.

The COVID-19 pandemic led to significant delays in cancer diagnosis and treatment, which has been confirmed in numerous studies [8]. Interruptions in healthcare systems including a reduction in the number of screenings performed and the cancellation or postponement of follow-up visits [5] contributed to many cancer cases being diagnosed at more advanced stages. As indicated by studies, these delays had a direct impact on worsening patient prognoses [8,9,10]. In the context of Poland, the COVID-19 pandemic also significantly affected cancer care [11,12,13]. Studies show that delays in diagnosis and treatment were widespread, negatively affecting patients. Furthermore, they indicate increased stress and uncertainty among cancer patients in Poland, which may have exacerbated the negative effects of the pandemic [14]. Poland’s healthcare system, like those in other countries, faced challenges related to hospital reorganizations and the limited availability of medical personnel [15]. Despite the implementation of telemedicine and other remote support forms, many patients had difficulty accessing cancer diagnostics and treatment [5,16].

Our research shows that the number of histopathological tests and cancer diagnoses in Lower Silesia changed significantly during and after the COVID-19 pandemic, consistent with reports from other regions around the world. However, our study provides new insights into regional differences in access to cancer diagnostics in Poland, taking into account the specific conditions of Lower Silesia. Notably, data from our region suggest that despite global downward trends, it is possible to partially restore the pre-pandemic diagnostic levels, which may result from effective regional healthcare management. Thus, this study highlights the need for regional epidemiological analyses, which can provide more precise data for future healthcare planning strategies.

One of the most concerning aspects of the pandemic was the increased mortality and morbidity among cancer patients. Studies have shown that delays in treatment and a later diagnosis of cancer lead to worse outcomes [17,18]. Moreover, an analysis found that the pandemic’s impact varied depending on the type of cancer, with some cancers being more vulnerable to the negative effects of treatment delays [19].

In response to the challenges posed by the pandemic, healthcare systems introduced a range of adaptations including the widespread use of telemedicine. The use of teleconsultations and remote patient monitoring allowed for the continuation of cancer care despite pandemic-related restrictions, as noted in other studies [20].

The pandemic exposed and exacerbated existing inequalities in access to cancer care. According to studies, patients from lower socioeconomic backgrounds and those living in regions with limited access to medical services were particularly vulnerable to the pandemic’s negative effects [21,22,23]. Globally, disparities in access to cancer care have become even more visible, according to the study [24].

The analysis of prostate cancer diagnostic trends in our study revealed significant disruptions caused by the COVID-19 pandemic, with both immediate and lasting implications for healthcare systems. Before the pandemic, diagnostic rates were consistent, reflecting the stability of routine screening and testing practices. However, during the pandemic, the data demonstrated a marked decline in the number of histopathological tests performed, particularly during the initial wave (March–June 2020), when testing levels reached their lowest point. This decrease was likely driven by limited access to healthcare services, patient reluctance to seek medical attention, and the reallocation of medical resources to address the pandemic.

Interestingly, despite the overall reduction in the number of tests, the frequency of positive diagnoses for prostate cancer increased during the pandemic (86.0%) compared to the pre-pandemic period (81.2%). This finding may indicate a shift toward more targeted testing, as only higher-risk cases were likely prioritized for histopathological assessment when healthcare resources were constrained. The slight increase in diagnostic frequency in the post-pandemic period (87.6%) further supports this hypothesis. While this approach may enhance resource efficiency and diagnostic precision, it also raises concerns about potential delays in identifying early-stage or asymptomatic cases that might have been detected through broader, routine screening.

The PSA test played a pivotal role during the pandemic, enabling healthcare systems to adapt more effectively to constraints. As a non-invasive blood test, the PSA test allowed for continued monitoring of prostate cancer risks, even when access to more invasive diagnostic procedures was restricted. This underscores the importance of flexible and scalable screening systems that can maintain essential diagnostic functions during healthcare crises. The relatively swift rebound in prostate cancer diagnoses post-pandemic highlights the resilience of the screening infrastructure in the studied region, suggesting that well-implemented systems can mitigate some of the long-term consequences of diagnostic delays.

Before the pandemic, the diagnostic workflow for breast cancer was stable, with a consistent number of histopathological tests and diagnoses. The frequency of positive diagnoses (46.85%) reflects the effectiveness of regular screening programs, such as mammography, in facilitating timely detection. During the pandemic, the number of tests performed dropped significantly, reflecting the strain on healthcare systems and the limitations in accessing routine medical services. Restrictions, the reallocation of healthcare resources, and patient concerns about contracting the virus led to a marked decline in testing. This decrease can be attributed to pandemic-related restrictions including the reprioritization of healthcare resources, reduced access to screening, and patient reluctance to seek medical care due to fear of infection.

Interestingly, despite fewer tests being conducted, the frequency of positive diagnoses increased significantly during the pandemic (65.23%). This trend may reflect a shift in diagnostic priorities, with clinicians focusing on symptomatic or higher-risk cases. It also underscores the potential underdiagnosis of asymptomatic or early-stage breast cancer cases, which may have contributed to delayed detection and potentially worsened outcomes for some patients.

In the post-pandemic period, there was a partial recovery in diagnostic activity, though it remained below the pre-pandemic levels. The continued elevated frequency of positive diagnoses may reflect the prioritization of high-risk cases and the need to address a backlog of missed or delayed diagnoses. Importantly, the recovery in breast cancer diagnostics was slower compared to other areas of healthcare. This is particularly concerning because delays in diagnosis can worsen patient outcomes by increasing the likelihood of late-stage presentations.

In the case of breast cancer, where access to mammography was notably disrupted, these delays highlight the need for strategies to improve early detection. Strengthening education on breast self-examination and developing more accessible diagnostic methods are crucial steps. These approaches can empower patients to recognize potential symptoms early and ensure timely medical evaluation, particularly during periods of systemic disruption.

The pandemic caused a significant decline in the number of histopathological examinations for colorectal cancer and adenomas, particularly during the first wave in 2020. The reduction in diagnostic activity was accompanied by a sharp drop in colorectal cancer and adenoma diagnoses, reflecting the challenges healthcare systems faced in maintaining essential services. Colonoscopies, being invasive and resource-intensive, were especially difficult to perform during this time, which contributed to delays in diagnosis. Alternative screening methods, such as fecal occult blood and DNA tests, were more frequently used but proved less effective in detecting cancers, emphasizing the need for new, minimally invasive screening approaches suitable for crisis situations.

The frequency of cancer or adenoma diagnoses remained relatively stable across the study periods.

Post-pandemic, diagnostic activity began to recover, with cancer diagnoses returning to near pre-pandemic levels, though adenoma diagnoses remained slightly lower. Interestingly, the frequency of positive diagnoses decreased after the pandemic, possibly reflecting a shift toward screening less severe or asymptomatic cases as healthcare systems resumed regular operations.

Statistical analysis confirmed the pandemic’s significant impact on diagnostic activity, with notable differences in colorectal cancer diagnoses between the pre-pandemic, pandemic, and post-pandemic periods. These findings underscore the importance of regular screening methods like colonoscopies for early detection as well as the need for more adaptable screening strategies to ensure continuity of care during healthcare crises.

The COVID-19 pandemic had a significant impact on cancer care, causing delays in diagnosis and treatment, worsening health outcomes, and increasing inequalities in access to care. These disruptions exposed vulnerabilities in healthcare systems, underscoring the need for resilience to better withstand future crises. While adaptations such as telemedicine mitigated some of the negative effects, they were not sufficient to fully address the challenges posed by the pandemic [20].

Our study highlights the pandemic’s impact on cancer diagnostics, with prostate cancer serving as a case study for the role of screening system resilience. Prostate cancer diagnostics, supported by the PSA test, experienced milder delays compared to other cancer types. In the studied region, the number of prostate cancer diagnoses returned to near pre-pandemic levels relatively quickly, reflecting the importance of well-designed and adaptable screening programs. Such systems help mitigate long-term consequences by reducing the number of undiagnosed cases and ensuring continuity of care, even under strained healthcare conditions.

The PSA test exemplifies how accessible and minimally invasive diagnostic tools can maintain diagnostic capacity in crisis situations [25]. For other cancers, the lack of similarly accessible tests resulted in more significant diagnostic challenges. For instance, breast cancer diagnostics were hampered by the limited availability of mammograms, increasing the importance of educating women on self-examination [26]. In colorectal cancer, colonoscopies became difficult to perform, and alternative diagnostic methods, such as fecal DNA and occult blood tests, proved less effective compared to the PSA test for prostate cancer [27]. These challenges underscore the need for continued innovation in diagnostic technologies, such as molecular tests and remote diagnostic tools, which could improve early cancer detection while reducing the reliance on invasive procedures and access to medical facilities.

Looking ahead, it is essential to continue studying the pandemic’s long-term effects on cancer patients and to refine health policies to minimize risks during future crises. Optimizing screening programs to balance efficiency and accessibility will be key to ensuring timely and accurate diagnoses, regardless of external challenges. Strengthening healthcare systems based on these lessons will help safeguard patient outcomes and reduce inequalities in access to care in the face of future global health emergencies.

## 5. Conclusions

The findings of this study clearly demonstrate the profound impact of the COVID-19 pandemic on cancer diagnostics in Lower Silesia, particularly for prostate, breast, and colorectal cancers. During the pandemic, a significant reduction in the number of histopathological examinations was observed, especially during the first wave, likely leading to delays in the early detection of cancers. Despite the reduced diagnostic activity, the frequency of positive diagnoses increased, suggesting a prioritization of high-risk cases. While this approach optimized resource allocation, it may have contributed to the underdiagnosis of asymptomatic or early-stage cancers.

Post-pandemic, the diagnostic activity partially recovered, but did not return to the pre-pandemic levels, with breast cancer diagnostics showing the slowest recovery, which could negatively affect patient outcomes. Importantly, the study revealed varied impacts depending on the type of cancer, highlighting the need for flexible healthcare management strategies during crises.

These results emphasize the importance of developing resilient healthcare systems equipped with minimally invasive diagnostic tools capable of operating effectively during disruptions. Additionally, the pandemic exposed disparities in access to healthcare, underscoring the necessity of addressing inequalities to improve outcomes and ensure equitable access to care. Future efforts should focus on long-term analyses of the pandemic’s effects, strengthening healthcare infrastructure, and implementing adaptive protocols to safeguard diagnostic continuity during future global health emergencies.

## Figures and Tables

**Table 1:** Average number of prostate cancer histopathological examinations performed per month (divided into positive diagnoses and non-cancerous changes) before, during, and after the COVID-19 pandemic.

	Tests Performed	Positive Diagnoses	Non-Cancerous Changes	Frequency of Positive Diagnoses
Before the pandemic (January 2018–February 2020)	49.85	40.46	9.38	81.2%
During the pandemic (March 2020–May 2022)	35.11	30.19	4.93	86.0%
The first wave of the pandemic (March–June 2020)	17.00	14.75	2.25	86.8%
After the pandemic (June–December 2022)	42.71	37.43	5.29	87.6%

**Table 2:** Average number of breast cancer histopathological examinations performed per month (divided into positive diagnoses and non-cancerous changes) before, during, and after the COVID-19 pandemic.

	Tests Performed	Positive Diagnoses	Non-Cancerous Changes	Frequency of Positive Diagnoses
Before the pandemic (January 2018–February 2020)	67.23	31.50	35.73	46.85%
During the pandemic (March 2020–May 2022)	37.93	24.74	13.19	65.23%
The first wave of the pandemic (March–June 2020)	24.50	15.75	8.75	64.29%
After the pandemic (June–December 2022)	44.71	29.29	15.43	65.50%

**Table 3:** Average number of colorectal cancer histopathological examinations performed per month (divided into colorectal and adenoma diagnoses and non-cancerous changes) before, during, and after the COVID-19 pandemic.

	Tests Performed	Diagnosed Colorectal Cancer	Diagnosed Colorectal Adenoma	Non-Cancerous Changes	Frequency of Diagnosis of Cancer or Adenoma
Before the pandemic (January 2018–February 2020)	305.73	66.35	146.50	92.88	69.62%
During the pandemic (March 2020–May 2022)	197.85	44.04	89.74	64.07	67.62%
The first wave of the pandemic (March–June 2020)	107.50	25.25	50.50	31.75	70.47%
After the pandemic (June–December 2022)	287.86	67.57	122.71	97.57	66.10%

## Data Availability

Data are available after email contact with the corresponding author.

## References

[1] Bray F., Ferlay J., Soerjomataram I., Siegel R.L., Torre L.A., Jemal A. (2018). Global Cancer Statistics 2018: GLOBOCAN Estimates of Incidence and Mortality Worldwide for 36 Cancers in 185 Countries. CA Cancer J. Clin..

[2] Pulumati A., Pulumati A., Dwarakanath B.S., Verma A., Papineni R.V.L. (2023). Technological Advancements in Cancer Diagnostics: Improvements and Limitations. Cancer Rep..

[3] Sung H., Ferlay J., Siegel R.L., Laversanne M., Soerjomataram I., Jemal A., Bray F. (2021). Global Cancer Statistics 2020: GLOBOCAN Estimates of Incidence and Mortality Worldwide for 36 Cancers in 185 Countries. CA Cancer J. Clin..

[4] Szkudlarek D., Gębarowski T., Hauzer N., Wiatrak B. (2024). The Concept of Health Debt Incurred during the COVID-19 Pandemic on the Example of Basal Cell Skin Cancer Diagnosis in Lower Silesia. J. Clin. Med..

[5] Grosman-Dziewiszek P., Wiatrak B., Jęśkowiak I., Szelag A. (2021). Patients’ Habits and the Role of Pharmacists and Telemedicine as Elements of a Modern Health Care System during the COVID-19 Pandemic. J. Clin. Med..

[6] Maringe C., Spicer J., Morris M., Purushotham A., Nolte E., Sullivan R., Rachet B., Aggarwal A. (2020). The Impact of the COVID-19 Pandemic on Cancer Deaths Due to Delays in Diagnosis in England, UK: A National, Population-Based, Modelling Study. Lancet Oncol..

[7] Kumar P., Rangaiah P., Augustine R. (2023). Improving Liver Cancer Diagnosis: A Multifaceted Approach to Automated Liver Tumor Identification in Ultrasound Scans. SSRN.

[8] Sud A., Torr B., Jones M.E., Broggio J., Scott S., Loveday C., Garrett A., Gronthoud F., Nicol D.L., Jhanji S. (2020). Effect of Delays in the 2-Week-Wait Cancer Referral Pathway during the COVID-19 Pandemic on Cancer Survival in the UK: A Modelling Study. Lancet Oncol..

[9] Patt D., Gordan L., Diaz M., Okon T., Grady L., Harmison M., Markward N., Sullivan M., Peng J., Zhou A. (2020). Impact of COVID-19 on Cancer Care: How the Pandemic Is Delaying Cancer Diagnosis and Treatment for American Seniors. JCO Clin. Cancer Inform..

[10] Wilkinson A.N. (2022). Mitigating COVID-19’s Impact on Missed and Delayed Cancer Diagnoses. Can. Fam. Physician.

[11] Kotrych D., Ciechanowicz D., Pawlik J., Szyjkowska M., Kwapisz B., Mądry M. (2022). Delay in Diagnosis and Treatment of Primary Bone Tumors during COVID-19 Pandemic in Poland. Cancers.

[12] Choręza P., Owczarek A.J., Kruk W., Chudek J. (2024). The Epidemiology of the Most Frequent Cancers in Poland in 2015–2021 and the Impact of the COVID-19 Pandemic on Cancer Incidence. Arch. Public Health.

[13] Trojanowski M., Radomyski P., Kycler W., Michalek I.M. (2024). The Impact of the COVID-19 Pandemic on Incidence Gap in Screen-Detectable Cancers: A Cohort Study in Greater Poland, Poland. Rep. Pract. Oncol. Radiother..

[14] Grajek M., Działach E., Buczkowska M., Górski M., Nowara E. (2021). Feelings Related to the COVID-19 Pandemic Among Patients Treated in the Oncology Clinics (Poland). Front. Psychol..

[15] Grosman-Dziewiszek P., Jęśkowiak-Kossakowska I., Szeląg A., Wiatrak B. (2024). Patterns of Dietary Supplement Use during the COVID-19 Pandemic in Poland: Focus on Vitamin D and Magnesium. Nutrients.

[16] Staszuk A., Wiatrak B., Tadeusiewicz R., Karuga-Kuźniewska E., Rybak Z. (2016). Telerehabilitation Approach for Patients with Hand Impairment. Acta Bioeng Biomech.

[17] Hartman H.E., Sun Y., Devasia T.P., Chase E.C., Jairath N.K., Dess R.T., Jackson W.C., Morris E., Li P., Hochstedler K.A. (2020). Integrated Survival Estimates for Cancer Treatment Delay Among Adults With Cancer During the COVID-19 Pandemic. JAMA Oncol..

[18] Cieślikowski W.A., Kasperczak M., Milecki T., Antczak A. (2023). Reasons behind the Delayed Diagnosis of Testicular Cancer: A Retrospective Analysis. Int. J. Environ. Res. Public Health.

[19] Tan Y.Y., Chang W.H., Katsoulis M., Denaxas S., King K.C., Cox M.P., Davie C., Balloux F., Lai A.G. (2024). Impact of the COVID-19 Pandemic on Health-Care Use among Patients with Cancer in England, UK: A Comprehensive Phase-by-Phase Time-Series Analysis across Attendance Types for 38 Cancers. Lancet Digit. Health.

[20] Bouabida K., Lebouché B., Pomey M.P. (2022). Telehealth and COVID-19 Pandemic: An Overview of the Telehealth Use, Advantages, Challenges, and Opportunities during COVID-19 Pandemic. Healthcare.

[21] Yoo K.J., Lee Y., Lee S., Friebel R., Shin S.a., Lee T., Bishai D. (2023). The Road to Recovery: Impact of COVID-19 on Healthcare Utilization in South Korea in 2016–2022 Using an Interrupted Time-Series Analysis. Lancet Reg. Health West Pac..

[22] Linjawi M., Shakoor H., Hilary S., Ali H.I., Al-Dhaheri A.S., Ismail L.C., Apostolopoulos V., Stojanovska L. (2023). Cancer Patients during COVID-19 Pandemic: A Mini-Review. Healthcare.

[23] Kim M., Park J.A., Cha H., Lee W.H., Hong S.N., Kim D.W. (2022). Impact of the COVID-19 and Socioeconomic Status on Access to Care for Otorhinolaryngology Patients. Int. J. Environ. Res. Public Health.

[24] Unger J.M. (2022). Cancer Care During COVID-19—A Shock to the System. JAMA Netw. Open.

[25] Van Poppel H., Albreht T., Basu P., Hogenhout R., Collen S., Roobol M. (2022). Serum PSA-Based Early Detection of Prostate Cancer in Europe and Globally: Past, Present and Future. Nat. Rev. Urol..

[26] Conte L., De Nunzio G., Lupo R., Mieli M., Lezzi A., Vitale E., Carriero M.C., Calabrò A., Carvello M., Rubbi I. (2023). Breast Cancer Prevention: The Key Role of Population Screening, Breast Self-Examination (BSE) and Technological Tools. Survey of Italian Women. J. Cancer Educ..

[27] Cubiella J., Calderón-Cruz B., Almazán R., Gómez-Amorín Á. (2023). Impact of the COVID-19 Pandemic on the Diagnosis of Colorectal Cancer within a Population-Based Organized Screening Program. Cancers.

